# Polymorphism of Leukocyte and Erythrocyte Antigens in Chronic Kidney Disease Patients in Southern Brazil

**DOI:** 10.1371/journal.pone.0084456

**Published:** 2014-01-07

**Authors:** Roger Haruki Yamakawa, Patricia Keiko Saito, Waldir Veríssimo da Silva Junior, Luiz Carlos de Mattos, Sueli Donizete Borelli

**Affiliations:** 1 Departamento de Ciências Básicas da Saúde, Universidade Estadual de Maringá, Maringá, Paraná, Brazil; 2 Departamento de Estatística, Universidade Estadual de Maringá, Maringá, Paraná, Brazil; 3 Departamento de Biologia Molecular, Faculdade de Medicina de São José do Rio Preto, São José do Rio Preto, São Paulo, Brazil; Centre for Inflammation Research, United Kingdom

## Abstract

We investigated the polymorphism of human leukocyte antigens (HLA) and Duffy erythrocyte antigens in chronic kidney disease (CKD) patients in southern Brazil. One hundred and eighty-three CKD patients, over 18 years old, on hemodialysis, were included. HLA-A, -B and -DRB1 typing was performed using the LABType®SSO (One Lambda, Inc.). Duffy phenotypes were determined by gel column agglutination using anti-Fy^a^ and anti-Fy^b^ monoclonal anti-sera. The patients' predominant ages ranged between 51 and 70 years (43%) and the predominant gender, ethnic group and dialysis period were, respectively, male (62%), white (62%) and 1–3 years (40%). The highest and lowest frequencies of Duffy phenotypes were Fy(a+b+) and Fy(a−b−), respectively. Nineteen HLA-A, 30 HLA-B and 13 HLA-DRB1 allele groups were identified. The most frequent HLA allele groups were HLA-A*01, -A*02, -A*03, -A*11, -A*24; HLA-B*07, -B*15, -B*35, -B*44, -B*51; HLA-DRB1*03, -DRB1*04, -DRB1*07, -DRB1*11 and -DRB1*13. Statistically significant differences were observed in the Duffy and HLA polymorphisms compared between CKD patients and healthy subjects. The Fy(a+b−) phenotype (p<0.0001, OR = 2.56, 95% CI = 1.60–4.07) was the most frequent in the patients (p<0.05), and the Fy(a+b+) phenotype (p = 0.0039, OR = 1.71, 95% CI = 1.18–2.51) was the most frequent in the healthy subjects in the same region of Paraná state (p<0.05). Regarding HLA, the HLA-B*42, -B*45, -B*51 and -DRB1*03 allele groups were the most frequent in the patients (p<0.05), and the HLA-B*44 allele group was the most frequent in the healthy subjects in the same region of Brazil (p<0.05). The polymorphism of these two markers among CKD patients in southern Brazil and healthy subjects of other studies, suggests that these markers might be involved with CKD development. Further studies should be undertaken to analyze the markers' influence on CKD and the long-term results from kidney transplantation.

## Introduction

The human leukocyte antigen (HLA) system is composed of molecules found on the surface of leukocytes and in almost all tissue cells. Its genes are highly polymorphic and co-dominant [1]. Several studies have reported the involvement of HLA alleles in many diseases [2], [3]. The HLA system has an important role in kidney transplants, since HLA compatibility between donor and recipient is extremely important to prevent organ rejection [4].

Besides the HLA system, other human systems are also involved in the analysis of compatibility between donor and recipient. Although the erythrocyte systems (ABO and Rh are the best known and the most important) are important parameters in transfusion and transplants, the role of the Duffy erythrocyte system is widely discussed because of its association with immediate and late hemolytic transfusion reactions, hemolytic disease of newborn children [5] and organ rejection processes [6], [7].

Because of the importance of the Duffy and HLA systems, several studies have delineated the erythrocyte and leukocyte profiles of healthy populations in different regions of the world [8], [9], [10], [11], [12]. However, very few studies have determined the profile of these systems in chronic kidney disease (CKD) patients. Since this is a chronic disease and its patients are possible transplant recipients, the present investigation evaluated the polymorphism of HLA and Duffy erythrocyte antigens in CKD patients in a population in southern Brazil.

## Materials and Methods

### Patients

The study included 183 CKD patients, of whom 114 were male and 69 female, over 18 years old (mean age 53±15 years). The CKD patients were of mixed ethnic origin, but predominantly white (n = 114). All were on hemodialysis in January through March 2011, in the city of Maringá in northwestern Paraná state, southern Brazil.

### Collection of data

The patients' age, gender, ethnic group and period of dialysis were retrieved from their medical records in hemodialysis clinics. The criterion for ethnic determination was self-defined ethnicity, based on the method used by the Brazilian Institute of Geography and Statistics (IBGE) national census survey (the official census of Brazil). This method classifies individuals in only a few pre-established color categories, which are based on self-classification and skin color, i.e. “Branco” (White), “Pardo” (Brown), “Preto” (Black), “Amarelo” (Yellow) and “Indígena” (Indigenous).

### HLA typing

About 5 mL of blood was collected from each patient in Vacutainer tubes (Becton and Dickson, Oxford, UK) with ethylenediaminetetraacetic acid (EDTA) as anticoagulant. The buffy coat was removed and the DNA genome extracted using the PureLink™ purification system (Invitrogen, Life Technologies, Carlsbad, NM, USA). LABType®SSO loci -A, -B and -DRB1 (One Lambda Inc., Canoga Park, CA, USA) were employed for the HLA typing. The protocol comprised the DNA amplification process, hybridization, reading on a special device (LABScan™100) and interpretation by software (HLA Fusion™). All procedures were performed according to the manufacturers' instructions.

### Duffy typing

Duffy erythrocyte phenotypes were defined by the gel column agglutination method (Diamed Latin America, Lagoa Santa, MG, Brazil), following the manufacturer's instructions. A 3% suspension of red blood cells for each sample, prepared in an isotonic diluent provided by the manufacturer, was added to gel card microtubes with anti-Fy^a^ and anti-Fy^b^ monoclonal anti-serum (Diamed Latin America). Each gel card was centrifuged, and inspected for the presence or absence of agglutination.

### Comparison of the present results with published data

In the Duffy comparisons, this study used as control the data published by Guelsin et al. [10] since their study was carried out in the same geographic region, and we adopted the same criteria to enroll our patients. In the HLA comparisons, this study used as control the data published by Ruiz et al. [9] and Bortolotto et al. [12] since their studies were carried out in the same region of Brazil.

### Statistical analysis

The statistical analysis used Microsoft Excel 2007 and Statistica version 7.0. The comparison parameters were Fisher's exact test and the relative risk estimate evaluated by odds ratio (OR) at the 95% confidence interval (CI).

### Ethics

The study was approved by the Committee for Ethics in Research of the Universidade Estadual de Maringá (Process 18592). All procedures followed Resolution 196/1996 of the Brazilian Health Council, which rules on research work on humans. All procedures were explained to each subject, and written informed consent was obtained from each subject.

## Results

Table 1 provides the patients' overall characteristics (age, gender, ethnic group, dialysis period). Males were predominant, with 62% of the 183 patients under analysis; the white ethnic group predominated, with 62% of the total population; 40% of patients had been dialyzed for between 1 and 3 years.

**Table 1:** General characteristics of CKD patients in southern Brazil.

Variables	n (183)	f
**Age (years)**		
18–30	14	0.076503
31–50	65	0.355191
51–70	79	0.431694
Over 70	25	0.136612
**Gender**		
Female	69	0.377049
Male	114	0.622951
**Ethnic group**		
Yellow	5	0.027322
White	114	0.622951
Brown	42	0.229508
Black	22	0.120219
**Dialysis period (years)**		
Less than 1	41	0.224044
1 to 3	74	0.404372
4 to 6	38	0.20765
Over 6	30	0.163934

Table 2 shows the frequency of Duffy phenotypes in CKD patients and compares the frequency of Duffy phenotypes of CKD patients with other studies on healthy subjects in the same region of Paraná state [10], in São Paulo state, Brazil [8] and worldwide [11]. Whereas Fy(a+b+) was the most frequent phenotype in approximately 35% of the population, Fy(a−b−) was rare and occurred in only 5%.

**Table 2:** Frequency of Duffy phenotypes in CKD patients in southern Brazil compared with healthy subjects in other studies [8], [10], [11].

Duffy Phenotype	CKD patients (n = 183)		GUELSIN et al. (2011) (PR) (n = 400)			NOVARETTI et al. (2000) (SP) (n = 2462)			HOWES et al. (2011) (worldwide) (n = 50578)		
	n	f	f	p value	OR (95% CI)	f	p value	OR (95% CI)	f	p value	OR (95% CI)
Fy(a+b−)	49	0.27	0.125	<0.0001	2.56 (1.60;4.07)	0.17	0.0012	1.80 (1.25;2.56)	0.33	0.0594	-
Fy(a+b+)	64	0.35	0.48	0.0039	1.71 (1.18;2.51)	0.22	0.0001	1.91 (1.37;2.66)	0.30	0.1452	-
Fy(a−b+)	60	0.33	0.37	0.3521	-	0.28	0.2026	-	0.23	0.0019	1.67 (1.20;2.29)
Fy(a−b−)	10	0.05	0.025	0.0853	-	0.33	<0.0001	8.53 (4.49;18.20)	0.14	0.0004	2.81 (1.49;5.97)
**TOTAL**	183	1	0.99			0.99			0.99		

Abbreviations for states: PR, Paraná; SP, São Paulo.

The sample analyses identified 19 HLA-A, 30 HLA-B and 13 HLA-DRB1 allele groups. HLA-A*02, -A*03 and -A*24 allele groups had the highest frequency for locus A. The most frequent allele groups in locus B were HLA-B*15, -B*35 and -B*51, whereas HLA-DRB1*03, -DRB1*04, and -DRB1*11 allele groups were the most common in locus DRB1. Table 3 shows the frequency of the HLA allele groups.

**Table 3:** Frequencies of HLA-A, -B and -DRB1 allele groups in CKD patients in southern Brazil.

HLA-A*	n	f	HLA-B*	n	f	HLA-DRB1*	n	f
**01**	35	0.0956284	**07**	28	0.0765027	**01**	32	0.0874317
**02**	85	0.2322404	**08**	17	0.0464481	**03**	42	0.1147541
**03**	40	0.1092896	**13**	6	0.0163934	**04**	52	0.1420765
**11**	26	0.0710383	**14**	13	0.0355191	**07**	39	0.1065574
**23**	19	0.0519126	**15**	30	0.0819672	**08**	21	0.057377
**24**	43	0.1174863	**18**	21	0.057377	**09**	7	0.0191257
**25**	6	0.0163934	**27**	12	0.0327869	**10**	11	0.0300546
**26**	16	0.0437158	**35**	37	0.1010929	**11**	55	0.1502732
**29**	12	0.0327869	**37**	3	0.0081967	**12**	2	0.0054645
**30**	20	0.0546448	**38**	9	0.0245902	**13**	39	0.1065574
**31**	17	0.0464481	**39**	15	0.0409836	**14**	18	0.0491803
**32**	11	0.0300546	**40**	15	0.0409836	**15**	34	0.0928962
**33**	13	0.0355191	**41**	8	0.0218579	**16**	14	0.0382514
**34**	3	0.0081967	**42**	9	0.0245902	**17**	0	0
**36**	2	0.0054645	**44**	24	0.0655738			
**66**	1	0.0027322	**45**	12	0.0327869			
**68**	13	0.0355191	**47**	1	0.0027322			
**69**	1	0.0027322	**48**	2	0.0054645			
**74**	3	0.0081967	**49**	9	0.0245902			
**80**	0	0	**50**	10	0.0273224			
**43**	0	0	**51**	44	0.1202186			
			**52**	10	0.0273224			
			**53**	9	0.0245902			
			**54**	1	0.0027322			
			**55**	4	0.010929			
			**56**	1	0.0027322			
			**57**	7	0.0191257			
			**58**	6	0.0163934			
			**67**	2	0.0054645			
			**81**	1	0.0027322			
			**78**	0	0			
			**82**	0	0			
			**73**	0	0			
			**46**	0	0			

Table 4 compares the frequency of the 15 most common HLA allele groups in CKD patients with the frequency of HLA allele groups in healthy subjects in the states of Paraná [9] and Rio Grande do Sul [12], Brazil, investigated in other studies.

**Table 4:** Distributions of the most frequent HLA allele groups in CKD patients in southern Brazil, compared to healthy subjects in other studies [9], [12].

HLA Allele Groups	CKD Patients (n = 183)		RUIZ et al. (2005) (PR) (n = 3500)			BORTOLOTTO et al. (2012) (RS) (n = 5000)		
HLA-A*	n	f	f	p value	OR (95% CI)	f	p value	OR (95% CI)
**01**	35	0.095628	0.095	0.9273		0.101	0.7914	
**02**	85	0.23224	0.228	0.8481		0.278	0.0568	
**03**	40	0.10929	0.093	0.311		0.104	0.7275	
**11**	26	0.071038	0.052	0.1187		0.051	0.0922	
**24**	43	0.117486	0.104	0.43		0.103	0.3818	
HLA-B*								
**07**	28	0.076503	0.069	0.597		0.07	0.6027	
**15**	30	0.081967	0.07	0.4013		0.084	1	
**35**	37	0.101093	0.112	0.6089		0.125	0.1963	
**44**	24	0.065574	0.105	0.0134	1.67 (1.1;2.66)	0.12	0.0009	1.94 (1.28;3.09)
**51**	44	0.120219	0.085	0.0278	1.47 (1.04;2.05)	0.087	0.0309	1.43 (1.01;1.99)
HLA-DRB1*								
**03**	42	0.114754	0.073	0.0057	1.65 (1.15;2.31)	0.099	0.3277	
**04**	52	0.142077	0.12	0.2172		0.124	0.2953	
**07**	39	0.106557	0.12	0.5079		0.131	0.2051	
**11**	55	0.150273	0.125	0.1694		0.119	0.072	
**13**	39	0.106557	0.117	0.6159		0.137	0.1027	

Abbreviations for states: PR, Paraná; RS, Rio Grande do Sul.

## Discussion

Perhaps one of the most heterogeneous in the world, the Brazilian population is a mixture of ethnic groups [13]. Ethnic miscegenation makes the search for a donor with the best immunological compatibility a rather difficult task. Investigation of the distribution of histocompatibility antigens in a population provides an important source of information for organ transplantation purposes. However, very few studies have determined the polymorphism of Duffy and HLA antigens in CKD patients. This contribution reports the frequencies of Duffy phenotypes and HLA allele groups in CKD Brazilian patients.

The frequencies of the Duffy blood group system phenotypes reported in this study were mainly compared with those published by Guelsin et al. [10], since we adopted the same criteria used in that study. Our enrolled patients and the bone-marrow volunteer donors selected by Guelsin et al. [10] came from the same geographic area and showed the same pattern of ethnicity. Additional comparisons are made with other studies [8], [11].

The Duffy Fy(a+b+) phenotype was prevalent in almost 35% of the CKD patients analyzed here, a percentage close to those reported by other authors [10]. The study of Guelsin et al. [10] included a sample of 400 voluntary blood and bone-marrow donors from the Maringá city, male (n = 217) and female (n = 183), mean age of 31.16±11.34 years (20–42 years). The donors were of mixed ethnic origin, but predominantly of Caucasian descent. Guelsin et al. [10] recorded a 48% frequency for the Fy(a+b+) phenotype in their study on polymorphism of erythrocyte systems in healthy subjects among the population in Paraná state. Comparison of these results showed a statistically significant difference, which indicated a greater prevalence of the Fy(a+b+) phenotype in populations of healthy subjects (p = 0.0039; OR = 1.71, 95% CI = 1.18–2.51). The frequency of the Fy(a+b−) phenotype also showed a statistically significant difference when these studies were compared with CKD patients, i.e., two times more predominant in CKD patients (p<0.0001; OR = 2.56, 95% CI = 1.60–4.07) (Table 2). Since these two studies were conducted on subjects from the same region, the comparative data suggest a certain association between the Duffy phenotype and CKD.

In comparison with the study of Novaretti et al. [8], which included 2,462 voluntary blood donors, both genders, Caucasians (n = 834), mulattos (n = 827), and blacks (n = 801), the results of the current study showed statistically significant differences in almost all Duffy phenotypes, underscoring the difference reported when frequencies of the Fy(a−b−) phenotype were compared between healthy subjects and CKD patients (p<0.0001; OR = 8.53, 95% CI = 4.49–18.20) (Table 2). These differences may be related to the predominant ethnic group studied by Novaretti et al. [8], whose population was approximately 66% blacks and browns (mulattos), whereas most subjects (62%) in the present study declared themselves to be white (Caucasians) and only 35% black or brown (mulattos). Several studies have shown that the Fy(a−b−) phenotype is a characteristic of people of African descent, due to a mutation at position -33T>C of the allele FY*B-promotor region, featuring the absence of Fy^b^ antigen expression solely in erythrocytes and with no changes in the expression of this protein in other tissues [11], [14], [15]. The Fy(a−b−) phenotype is rarely detected in white populations. In fact, it is caused by specific mutations of the replacement-deletion type, with no occurrence of the protein Duffy expression in erythrocytes and in other tissues [16], [17], [18].

When Duffy phenotypes in the CKD patients were compared with data from the world survey by Howes et al. [11], which included a total of 50,578 samples from Africa (n = 11,370), Americas (n = 10,939), Asia (n = 11,143) and Europe (n = 17,126), statistically significant differences in the Fy(a−b+) and Fy(a−b−) phenotypes could be detected, showing a high frequency of the Fy(a−b+) phenotype in CKD patients compared with world population (p = 0.0019; OR = 1.67, 95% CI = 1.20–2.29). Low expression of the Duffy antigen is proportionally higher in the world population than in CKD patients. The above association was demonstrated by a higher frequency of the Fy(a−b−) phenotype in the world population (p = 0.0004; OR = 2.81, 95% CI = 1.49–5.97) (Table 2). Luo et al. [19] and Zarbock et al. [20] showed that rats knocked out for the Duffy antigen had a lower cell infiltration and a better kidney performance than those of wild ones in a local-injury model. The fault in the neutrophile movement was consequently associated with kidney protection.

Since the Duffy antigen may be found in non-erythroid cells [21], [22], [23], its expression on the cell surface may be a factor that affects the survival of a surgery graft. According to Lerut et al. [7], in their investigation on the Duffy system in kidney transplants, donor-receptor mismatches for the Duffy system showed more chronic lesions compared with donor-receptor matching grafts, suggesting that Duffy antigens may function as minor histocompatibility antigens. Further, Segerer et al. [6] reported an increase in expression of Duffy molecules in patients with cellular and humoral rejection coupled to a greater deposition of fraction C4d in peritubular capillaries.

The investigation of HLA polymorphism provides important data for genetic and population studies in every country [24]. Due to the high degree of ethnic miscegenation in the Brazilian population, the frequencies of HLA alleles may vary according to the predominant ethnic group in a locale [12], [24], [25]. Studies undertaken from samples of populations from all continents revealed differences among the frequencies of HLA alleles and showed them to be increased in mixed-race populations [26], [27]. However, a study comprising different samples from Brazilian populations, whites and blacks, showed that the similarities among HLA allele frequencies of these populations were greater than their differences [28]. These data concord with the current study.

The 15 most frequent HLA allele groups in the population studied here were HLA-A*01, -A*02, -A*03, -A*11, -A*24; HLA-B*07, -B*15, -B*35, -B*44, -B*51; HLA-DRB1*03, -DRB1*04, -DRB1*07, -DRB1*11 and -DRB1*13. These frequencies of the HLA allele groups were compared with those published by Ruiz et al. [9] and Bortolotto et al. [12]. Our enrolled patients and the volunteer bone-marrow donors selected by Ruiz et al. [9] and Bortolotto et al. [12] came from the same region of Brazil and showed the same ethnic composition, predominantly white.

The study of Ruiz et al. [9] included a sample of 3,500 healthy volunteer bone-marrow donors, including Caucasians (n = 2775), Orientals (n = 33) and Afro-Brazilians (n = 77). Additional groups were mulattos (n = 186, admixed individuals of predominantly African and European origin), cafuzos (n = 319, admixed individuals of predominantly African and Amerindian origin) and 110 individuals from the total sample had no ancestry group specified. The study of Bortolotto et al. [12] included a sample of 5,000 volunteer bone-marrow donors. Sixty-nine percent of the donors were women, and the donors' ages ranged between 18 and 54 years, including Caucasians (n = 4,428), blacks (n = 248), and mestizos (n = 324).

When the results for HLA frequencies of CKD patients were compared with those of healthy subjects in studies by Ruiz et al. [9] and Bortolotto et al. [12], we found great similarities among these populations (Table 4). Nevertheless, there were statistically significant differences between the HLA-B*42, -B*44, -B*45, -B*51 allele groups in this study and those found by Bortolotto et al. [12] and the HLA-B*44, -B*51, -DRB1*03 allele groups in this study and those found by Ruiz et al. [9]. The HLA-B*42, -B*45, -B*51 and -DRB1*03 allele groups were most frequent in the CKD patients (p<0.05), and HLA-B*44 was most frequent in the healthy subjects (p<0.05).

Due to the high degree of polymorphism, the HLA system has been studied as a genetic marker involved in susceptibility to several diseases [2], [3]. The HLA-A*74 and HLA-DRB1*11 allele groups showed a positive association with terminal CKD in a case-control study with a small group of CKD patients from southeastern Brazil [29]. HLA-Cw2 showed a negative association with end-stage renal disease, and HLA-DQB1*03(8) was positively associated with the risk of end-stage renal disease in a case-control study with 235 unrelated Saudi patients awaiting renal transplantation [30].

The present investigation was limited by the small number of patients evaluated. Because only a few clinics were included, not all patients receiving dialysis in the region were studied. Despite this limitation, the data were sufficient for statistical analysis and for the purpose of this study, and provides important information on leukocyte and erythrocyte antigens in Brazilian CKD patients.

## Conclusions

The frequencies of Duffy and HLA antigens vary among different populations. Comparison of the polymorphism of these two markers in southern Brazilian CKD patients and in healthy subjects from the same region and from others [8], [11] allow us to suggests that Duffy antigens and HLA allele groups may be involved in CKD. Further studies are required to analyze the influence of these markers on CKD and transplant evolution.
